# Development of a serological assay to predict antibody bactericidal activity against non-typeable *Haemophilus influenzae*

**DOI:** 10.1186/s12866-015-0420-x

**Published:** 2015-04-18

**Authors:** Giuseppe Ercoli, Buket Baddal, Greco Alessandra, Sara Marchi, Roberto Petracca, Beatrice Aricò, Mariagrazia Pizza, Marco Soriani, Silvia Rossi-Paccani

**Affiliations:** University of Leicester, Department of Genetics, Adrian building, Leicester, UK; Novartis Vaccines & Diagnostics s.r.l. (a GSK company), Via Fiorentina 1, 53100 Siena, Italy

**Keywords:** Non-typeable *Haemophilus influenzae*, Serum bactericidal assay, Antibody, Complement, Vaccine

## Abstract

**Background:**

Non-typeable *Haemophilus influenzae* (NTHi) is a Gram negative microorganism residing in the human nasopharyngeal mucosa and occasionally causing infections of both middle ear and lower respiratory airways. A broadly protective vaccine against NTHi has been a long-unmet medical need, as the high genetic variability of this bacterium has posed great challenges.

**Results:**

In this study, we developed a robust serum bactericidal assay (SBA) to optimize the selection of protective antigens against NTHi. SBA takes advantage of the complement-mediated lysis of bacterial cells and is a key *in vitro* method for measuring the functional activity of antibodies. As a proof of concept, we assessed the bactericidal activity of antibodies directed against antigens known to elicit a protective response, including protein D used as carrier protein in the Synflorix pneumococcal polysaccharide conjugate vaccine. Prior to SBA screening, the accessibility of antigens to antibodies and the capacity of the latter to induce C3 complement deposition was verified by flow cytometry. Using baby rabbit serum as a source of complement, the proposed assay not only confirmed the bactericidal activity of the antibodies against the selected vaccine candidates, but also showed a significant reproducibility.

**Conclusions:**

Considering the rapidity and cost-effectiveness of this novel SBA protocol, we conclude that it is likely to become an important tool to prove the capability of antibodies directed against recombinant antigens to induce NTHi *in vitro* killing and to both select new protective vaccine candidates, and predict vaccine efficacy.

**Electronic supplementary material:**

The online version of this article (doi:10.1186/s12866-015-0420-x) contains supplementary material, which is available to authorized users.

## Background

Non-typeable *Haemophilus influenzae* (NTHi) is a Gram negative microorganism that differs from the other Hi serotypes for the lack of a polysaccharide capsule. NTHi is a commensal in the human nasopharyngeal mucosa and may occasionally act as a pathogen initiating infections of both the upper and lower respiratory airways, causing conditions such as otitis media (OM), chronic obstructive pulmonary disease (COPD), and invasive diseases such as meningitis and sepsis, especially in neonates and the elderly [1-3].

A broadly protective vaccine against NTHi is a current medical need. Indeed, one of the major hurdles to NTHi vaccine development is the high genetic diversity among strains, leading to the necessity to identify antigens carrying functional epitopes able to induce cross-protective antibodies [4].

As NTHi does not express a capsule, the search for alternative vaccine candidates has so far been focused on outer membrane proteins and lipo-oligosaccharide [5]. A protective antigen should express epitopes available for antibody binding on the surface of the intact bacterium. Molecules embedded within the outer membrane or blocked by steric hindrance of adjacent structures, such as sialic acid could be unavailable for antibody binding and therefore unable to generate protective antibodies. Currently, major (P1, P2, P4, P5) and minor (P6, D15, TbpA/B) outer membrane proteins, LPS, and adhesins (HMW, Hia, pili, P5) are being studied, and preclinical results including the use of animal models have been generated. In particular, protective efficacy of PD, OMP26 and P5 was proved using models of otitis (chinchilla model of otitis media) and lung infection (rat model of pulmonary clearance) [6-8]. This approach provided important information on the impact of the host-pathogen relationship on whole tissue, and the interaction between humoral and cellular response. However, these experiments are very expensive, and consequently limit the possibility to screen for strains with different genetic background.

A well-established *in vitro* method to predict antigen protective ability is the Serum Bactericidal Assay (SBA). The SBA is also referred to as complement-mediated bacterial killing and quantifies bacterial cell death after incubation of bacterial cells with immune serum and a complement source. In particular for *Neisseria meningitidis,* SBA activity has been shown to highly correlate with immunity to meningococcal disease [9-11] and more recently Borrow’s group has evaluated and validated a novel SBA for *Haemophilus influenzae* type B [12]. However, SBA was never established for NTHi due to the high susceptibility of this non-encapsulated microorganism to complement-mediated killing. In this study, we modified and optimized SBA to evaluate the functional activity of antibodies generated following mice immunization with a panel of recombinant NTHi antigens already reported to have protective capability in animal models. This assay could constitute a rapid and low cost tool for antigen selection.

## Results and discussion

### Selection of the antigens to develop a Serum Bactericidal Assay for NTHi

The aim of this study was to set up a robust assay to screen the antigens capable of raising functional antibodies to kill NTHi. First of all, we selected from the literature a panel of proteins already reported to be protective in animal models (Protein E [13], Protein D [14], P6 [15], OMP26 [16], PilA and HtrA [17]). This helped us to set the best working conditions of the assay. Moreover, we used an unrelated protein (that is not present on NTHi surface) as negative control. We selected NHBA from *N.meningitidis* as unrelated protein-it is a surface protein that is strictly specific for meningococcus.

### Surface accessibility of NTHi antigens

**Figure 1:**
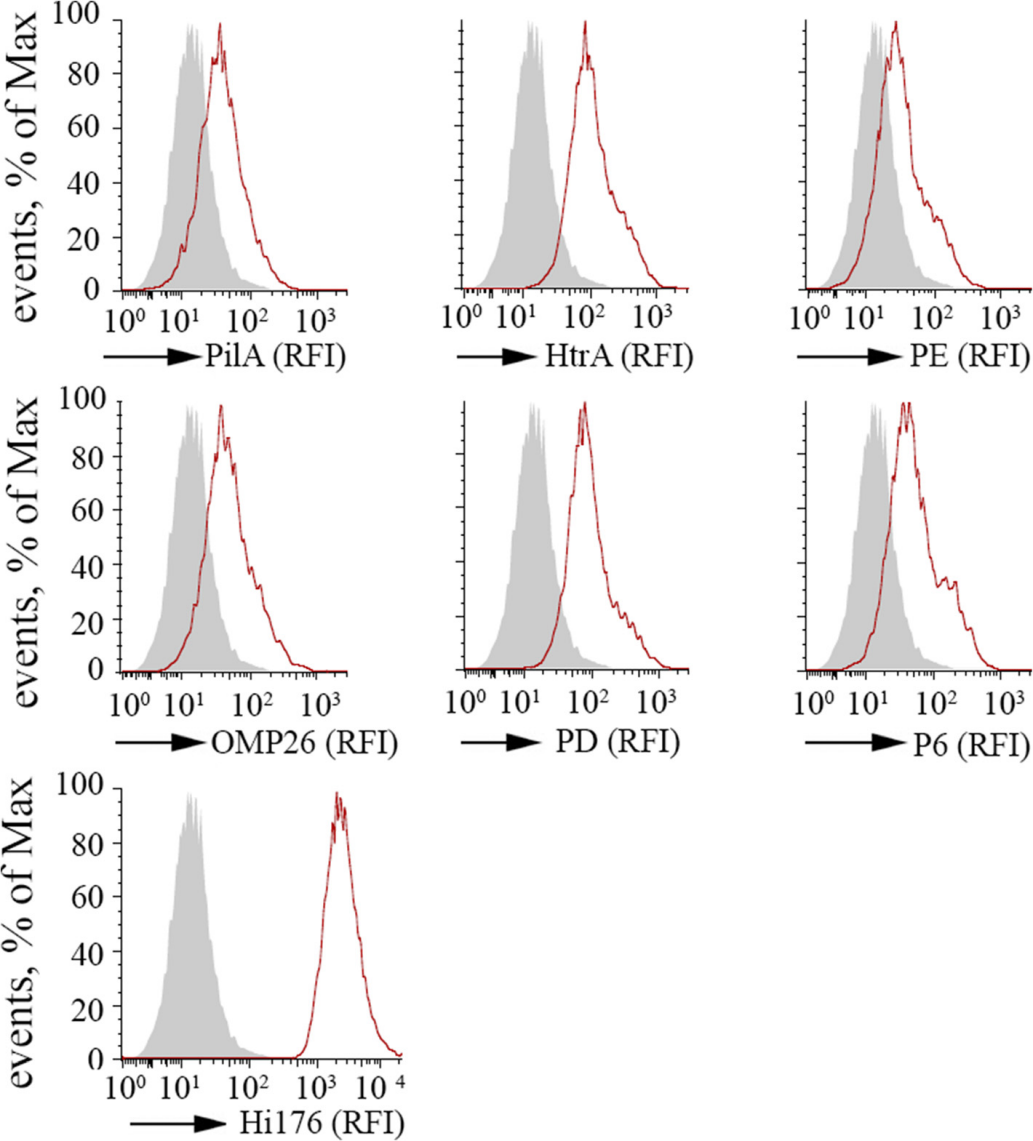
NTHi antigens are accessible to antibodies on bacterial surface. Binding of anti-PD, PE, P6, OMP26, HtrA, PilA and anti-176 to live bacteria was evaluated by flow cytometry. Histograms represent a typical experiment out of two performed with similar results. RFI, relative fluorescence intensity. Filled gray line, bacteria incubated with an irrelevant serum; red line, bacteria incubated with antigen specific serum.

Since the prerequisite for the functionality of the assay is that the antigen tested should be exposed on the bacterial surface, we verified the surface exposure of the selected antigens. First of all the antigens were formulated with AL(OH)_3_ as an adjuvant, and immunized in mice, after which polyclonal antibodies were obtained. As shown in Figure 1, all the antigens tested (PD, PE, P6, OMP26, HtrA and PilA) were well exposed on the surface of live bacteria.

### Selection of a complement source

**Figure 2:**
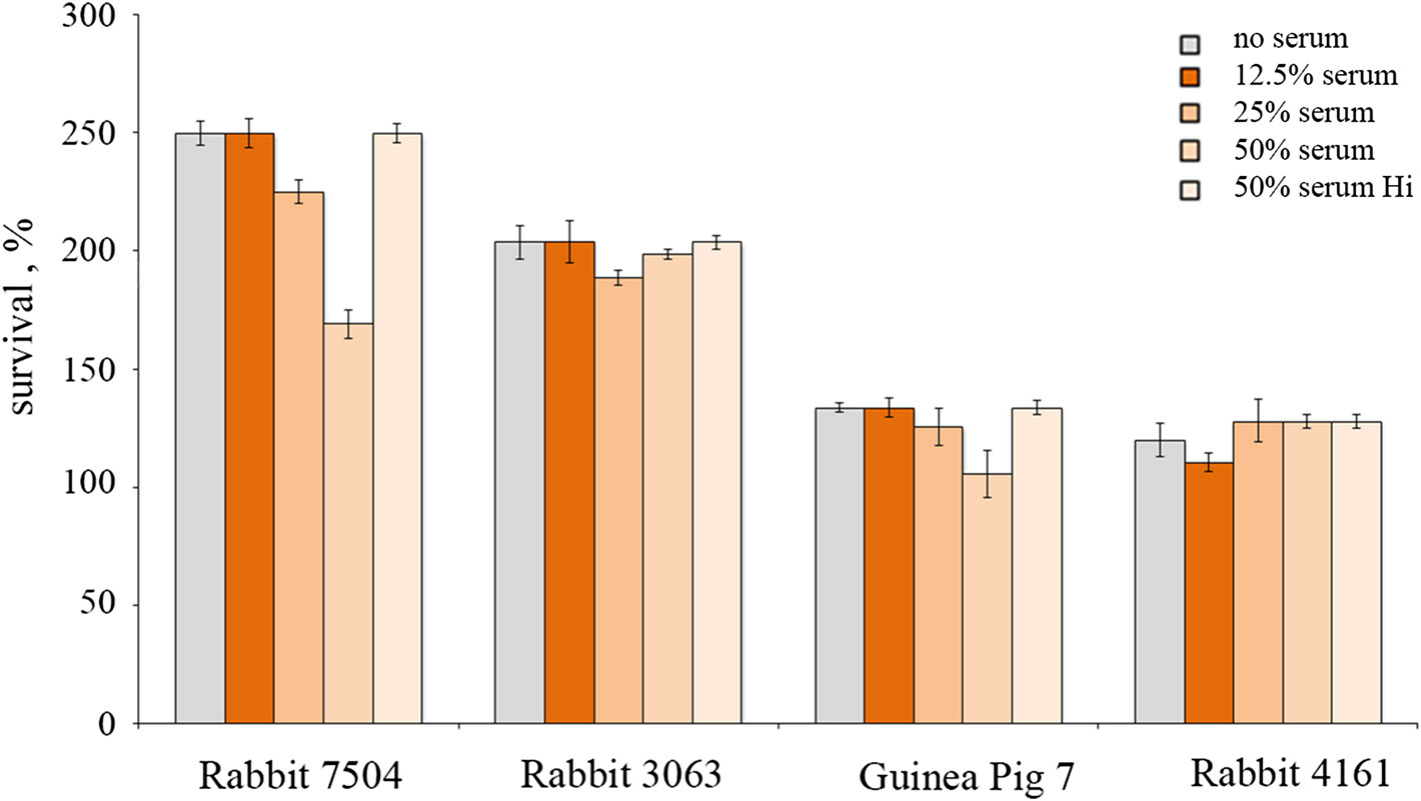
Selection of complement source. 176 strain was tested for survival at different baby rabbit or guinea pig serum concentrations (12.5%, 25%, 50%) for 60 min. Bacteria without complement and bacteria with heat-inactivated complement (dilution factor 50%) were used as negative control. The results are expressed as % survival at 60 min. The means and standard errors from 3 independent experiments are shown. Error bars represent the SD.

Selection of a suitable source of complement represented a crucial step in setting up a SBA. Several lots of sera derived from baby rabbit and guinea pigs were screened to evaluate toxicity. Briefly, 176 strain was incubated for 60 min with different concentration (12.5%, 25% 50%) of baby rabbit or guinea pig complement. Bacteria without complement and bacteria with heat-inactivated complement (dilution factor = 50%) were used as control. The mixture was then plated on agar chocolate plates to evaluate NTHi strain survival. We discarded sera which resulted to be toxic for NTHi, and identified Baby Rabbit serum (lot 7504) as good complement source for the assay (Figure 2). We assumed that a serum was not toxic at the dilution used in the SBA (25%), when the number of bacteria at T_60min_ was more than double the number of targets cells present before the incubation with complement.

### Evaluation of C3 complement deposition

**Figure 3:**
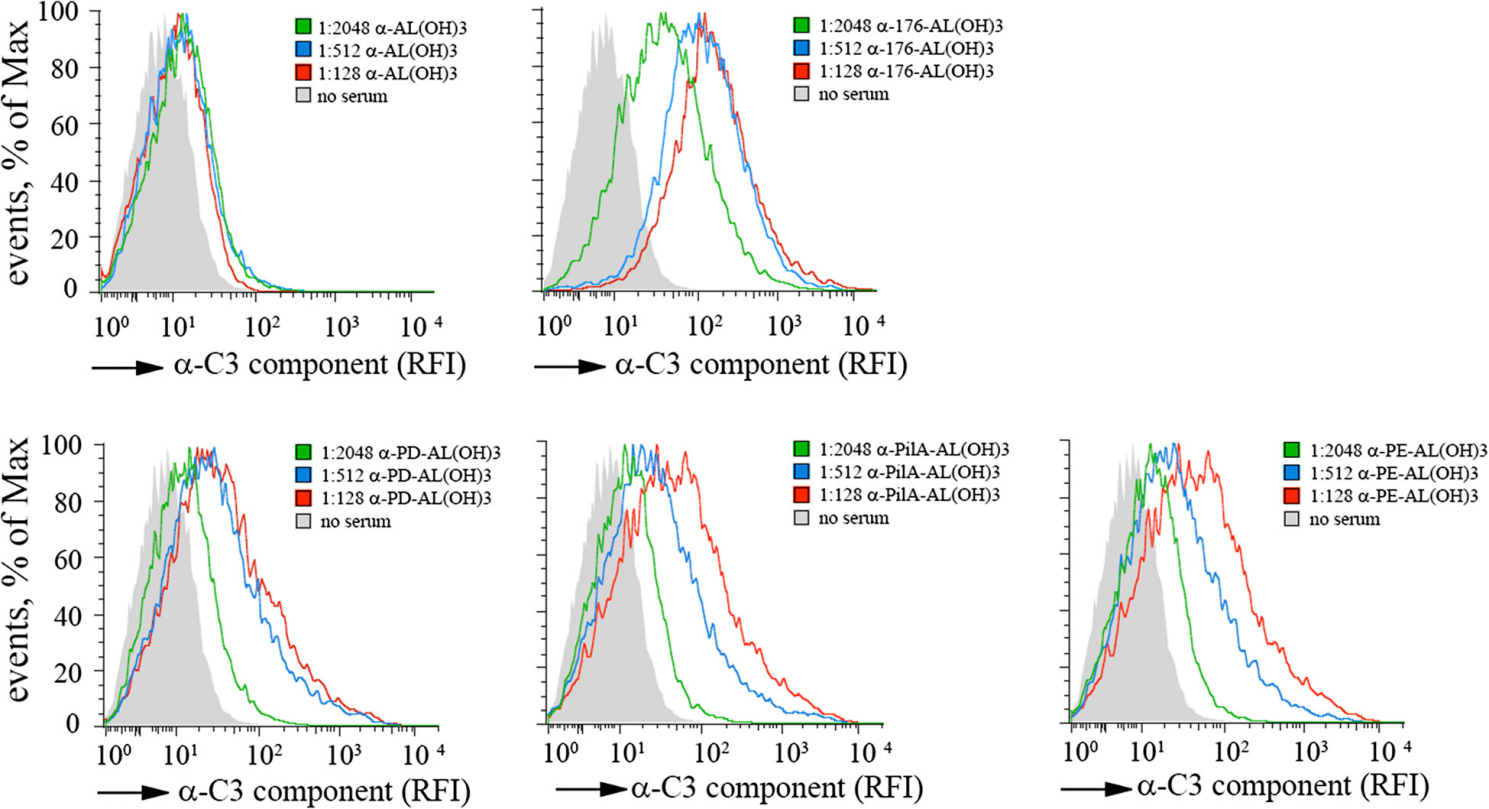
Sera against specific antigens promote C3 fragment deposition on NTHi. 176 strain was incubated with rabbit complement in the absence or presence of increasing amounts of sera from mice immunized with NTHi antigens or 176 strain heat inactivated. The amount of C3 deposited on bacteria (mean fluorescence) was measured by flow cytometry. Serum form mice given alum was used as negative control. Histogram tracings from one representative experiment for anti-176 serum are shown in addition to three of the antigens analyzed. RFI, relative fluorescence intensity.

We next assessed the ability of the specific sera to induce complement deposition on NTHi surface. Using flow cytometry analysis, we therefore evaluated if specific antibodies against NTHi antigens were able to promote C3 deposition, a crucial step in complement pathway cascade. Basically, bacteria in exponential phase were incubated with rabbit complement in the absence or presence of increasing amounts of sera from mice immunized with NTHi antigens (Figure 3, *lower panel*) or 176 strain heat inactivated (Figure 3, *upper panel*). C3 deposition was detected using anti-rabbit C3 antibody and anti-Rabbit Secondary Antibody, Alexa Fluor® 488 conjugate. As reported in Figure 3, anti-176 as well as anti-PilA, anti-PD and anti-PE antibodies were able to promote C3 component deposition on bacterial surface leading to activation of complement pathway. Serum form mice given alum was used as negative control.

### Development of a reproducible serum bactericidal assay

The functionality of the antibodies raised against 176 strain or against specific NTHi antigens was assessed by SBA. To perform the SBA, bacteria in exponential phase were incubated with complement in the absence or presence of increasing amounts of sera from mice immunized with selected antigens. To validate the assay, we evaluated bactericidal activity of antibodies directed against antigens proved to be protective in *in vivo* models of colonization and infection, such as Protein E [13], Protein D [14], P6 [15], OMP26 [16] and HtrA [17]. As postulated, sera raised against PD, PE, P6, OMP26, HtrA and PilA antigens were able to induce *in vitro* killing of 176 strain, consistently with their capacity to promote complement deposition. SBA titers obtained vary from protein to protein and it is due to several factors, including antigen density and antibody affinity. Notably, SBA titers well correlated with surface expression of antigens (Figure 1). In particular, PD and HtrA that appeared to be more accessible to antibodies on the bacterial surface, were among the antigens giving higher SBA titers (Table 1). Sera from a) non-immunized mice (pre-immune sera); b) mice immunized with PBS alone; c) mice immunized with adjuvant alone (AL(OH)_3_); d) mice immunized with an adjuvanted unrelated antigen (NHBA from *N. meningitidis*) were used as negative controls. None of these sera induced detectable SBA titers (Table 1).

**Table 1:** **Bactericidal activity against 176 strain**

**Sera**	**SBA titers**
**α-PilA-**AL(OH)3	341 ± 147
**α-HtrA-**AL(OH)3	853 ± 295
**α-PE-**AL(OH)3	512
**α-OMP26**-AL(OH)3	192 ± 90
**α-PD**-AL(OH)3	853 ± 295
**α-P6**-AL(OH)3	213 ± 74
**α-**AL(OH)3	<4
**α-176**	8192
**α-NHBA-**AL(OH)3	<16
**pre-immune sera**	<4
**α-PBS**	<4

Bactericidal titers obtained using sera against specific antigens are reported. Titers are expressed as the reciprocal of the serum dilution necessary to obtain >50% bacterial killing. Experiments were performed three times and average titers is reported. Serum against NTHi was used as positive control. Sera from non-immunized mice (pre-immune sera), mice immunized with PBS alone, mice immunized with adjuvant alone (AL(OH)_3_), mice immunized with an adjuvanted unrelated antigen (NHBA from *N. meningitidis*) were used as negative controls. Data are means and standard errors of the means from 3 separate experiments.

Furthermore, given the extensive genetic diversity exhibited by NTHi we validated the bactericidal assay with genetically diverse strains belonging to different clades identified by genome-based population structure of NTHi [4]. Preliminary data show that the assay is applicable also to strains genetically different (data not shown).

## Conclusions

Reverse Vaccinology and other profile-based computational approaches [18] allow the identification of a large number of open reading frame encoding for surface proteins, however the subsequent screening for bactericidal epitopes is a clear bottleneck. *In vitro* SBA has been proved to be a rapid and cost-effective method to identify new vaccine candidates and, as in the case of the Type B meningococcus vaccine, a valid correlate of protection [9-11]. A high genetic diversity not correlating with disease is an important signature of NTHi species and represents a crucial issue in the development of a successful vaccine against this human pathogen. Selection of protective antigens should therefore consider this aspect by supporting the prioritization of candidates carrying functional epitopes able to induce cross-protective antibodies. In this study, we propose a novel SBA for NTHi that would help to identify broadly-protective vaccine candidates through a rapid screening of both antigens and strains.

## Methods

### Antibodies and reagents

Antibody against C3 complement component was from Calbiochem. Goat anti-Mouse IgG (H + L) Secondary Antibody, Alexa Fluor® 488 conjugate, and Goat anti-Rabbit IgG (H + L) Secondary Antibody, Alexa Fluor® 488 conjugate were from Molecular Probes.

Baby rabbit complement and Guinea Pig complement were from Cederlane Labs. AL(OH)_3_ was purchased from Sigma-Aldrich.

### Bacterial strains and growth conditions

NTHi strain 176 (kindly provided by Richard Moxon and Derek Hood, Oxford University, UK), isolated from a child with otitis media, was used for this study. NTHi was grown on chocolate agar polivitex (BioMerieux) at 37°C with 5% CO_2_. Brain-heart infusion (BHI) broth (Difco Laboratories) supplemented with 10 μg/mL each of haemin (Fluka Biochemika) and nicotinamide adenine dinucleotide (NAD, Sigma) was used as fluid growth medium. *Escherichia coli* strains DH5α, HK100 and BL21 (DE3) (Invitrogen) were used for cloning and expression of NTHi antigens. They were cultured at 37°C in Luria Bertani (LB) medium and, when required, supplemented with 100 μg/mL ampicillin.

### Cloning of genes coding for vaccine antigens

NTHi genes were cloned into the pET15b + vector (Novagen) using the polymerase incomplete primer extension (PIPE) method [19]. Sequences coding for each protein were amplified by PCR from the *176* genomic DNA, removing the signal peptide (primers are listed in Additional file 1: Table S1 in the supplemental material). PCRs generated mixtures of incomplete extension products; short overlapping sequences were introduced at the ends of these incomplete extension mixtures, which allowed complementary strands to anneal and produce hybrid vector-insert combinations. *Escherichia coli* HK100 cells [20] were then transformed with vector-insert hybrids. Single ampicillin-resistant colonies were selected and checked for the presence of the recombinant plasmid by PCR. Plasmids from positive clones were isolated and subcloned into competent *E. coli* BL21(DE3) cells.

### Antigen expression, purification and formulation

For protein expression and purification, one single colony of *E. coli* BL21(DE3) strain expressing NTHI antigens was inoculated in LB plus ampicillin and grown overnight at 37°C, diluted in fresh LB medium and grown at 30°C to an OD_600_ of 0.6-0.8. Protein over-expression was induced by the addition of 1 mM isopropyl-1-thio-β-D-galactopyranoside (IPTG, Sigma) for 3 hours. Recombinant 6 x His-fusion proteins was purified by affinity chromatography on Ni^2+^-conjugated chelating fast-flow Sepharose 4B resin (Pharmacia). Purity was checked by SDS-PAGE electrophoresis staining with Coomassie blue. Protein concentration was determined using the bicinchoninic acid (BCA) assay (Thermo Scientific). Endotoxin content was assessed using the limulus amoebocyte lysis (LAL) test.

Antigens were adsorbed onto alum by incubating 10 μg of each antigen with 2 mg/ml aluminum hydroxide, with slow stirring for few hours at room temperature (RT). The pH and osmolality of the formulation and antigen absorption were determined.

### Polyclonal antisera production

In order to produce polyclonal antisera, groups of eight CD1 mice were immunized with 10 μg of each purified Ag adsorbed onto aluminum hydroxide. The recombinant protein was given intraperitoneally on day1. Booster doses were administered on day 21 and 35. Blood samples were taken on day 49. To generate anti-NTHi antibodies, mice were injected with heat inactivated bacteria (10^8^ cfu in each dose) following the same administration and sampling route. Control mice received equal amounts of saline or alum or adjuvanted unrelated antigen. All treatments were performed in accordance with internal animal ethical committee and institutional guidelines.

### Complement toxicity assay

Toxicity assay was performed in order to screen the best source of complement to use in the SBA. A panel of baby rabbit and guinea pig sera was tested to evaluate toxicity against NTHi. In particular, serially diluted baby rabbit or guinea pig sera were incubated with mid-log phase bacteria for 1 h at 37°C and the survival rate was calculated for each dilution by CFU counting. The selection of the ideal source of complement was based on two main criteria: the serum should show an activity proportional to its concentration and it should not be toxic for NTHi. We assumed that a serum was not toxic when the number of bacteria at T_1hour_ was more than double the number of target cells present before complement incubation. Heat inactivated sera were used as negative control.

### Flow cytometry analysis

Surface exposure of NTHi antigens was assessed by incubating bacteria in exponential phase with sera derived from mice immunized with recombinant proteins for 30 min at 4°C. After washing, cells were stained with anti-Mouse Secondary Antibody, Alexa Fluor® 488 conjugate. Cells were analyzed with a Canto II flow cytometer (Beckton-Dickinson) using Flowjo software. The geometric mean fluorescence intensity (MFI) for each population was calculated.

To assess C3 complement component deposition, bacteria in exponential phase were incubated with 25% baby rabbit serum, as a source of complement, plus serial dilution of specific polyclonal anti-sera for 20′ at 37°C, 180 rpm. The capacity of specific sera to promote C3 deposition was revealed using anti-rabbit C3 antibody and anti-Rabbit Secondary Antibody, Alexa Fluor® 488 conjugate. Cells were analyzed with a Canto II flow cytometer (Beckton-Dickinson) by using Flowjo software. The geometric mean fluorescence intensity (MFI) for each population was calculated.

### Serum bactericidal assay (SBA)

Non-typeable *Haemophilus influenzae* target strain lysis was assessed in the presence of NTHi-specific antibody and complement (antibody mediated, complement-dependent killing). Antisera were tested in a serum bactericidal assay to verify their capacity to induce NTHi *in vitro* killing. Sera from non-immunized mice (pre-immune sera), mice immunized with PBS alone, mice immunized with adjuvant alone (AL(OH)_3_) and mice immunized with an adjuvanted unrelated antigen (NHBA from *N. meningitidis*) were used as negative controls.

**Figure 4:**
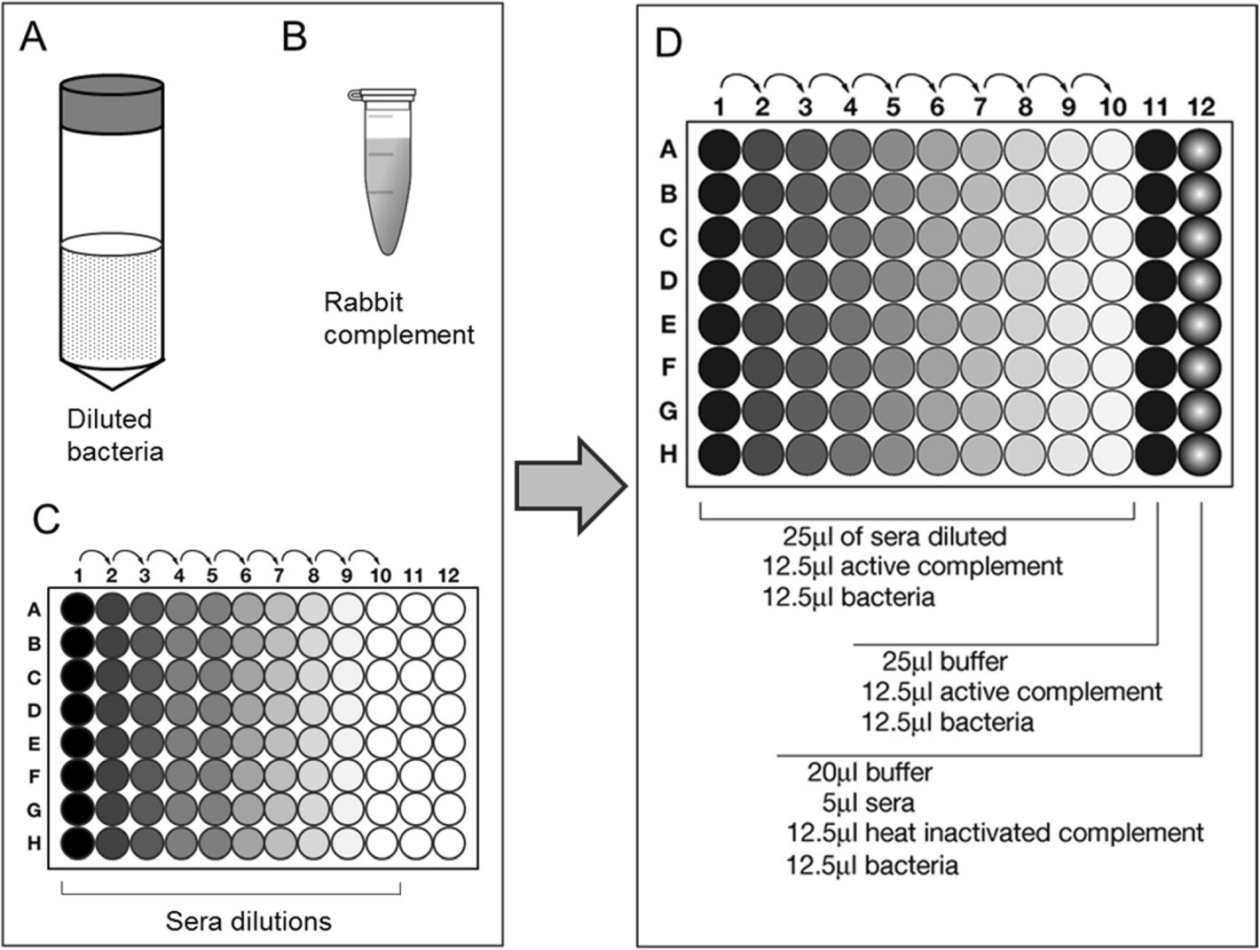
NTHi SBA protocol. Bacteria are diluted in Dulbecco’s PBS containing 1% (w/v) BSA and 0.1% (w/v) glucose **(A)**; they are then incubated with baby rabbit complement **(B)**, and serially diluted sera raised against the selected antigens **(C)**. The microtiter plate layout for the NTHi SBA assay is shown in panel **(D)**.

Bacteria were subcultured on chocolate round agar plates and incubated ca. 18 hours at 37°C, 5% CO_2._ Colonies were inoculated in 4 ml of BHI plus hemin (10 μg/ml) and NAD (10 μg/ml) and incubated on a shaker at 37°C with 5% CO_2_ 180 rpm until OD_600_ = 0.5 (6x10^8^ CFU/ml). Bacteria were centrifuged for 10 minutes, 2000 x g, at room temperature. Supernatants were discarded and the pellet was resuspended in Dulbecco’s PBS containing 1% (w/v) BSA and 0.1% (w/v) glucose (*assay buffer*). A working dilution of bacteria was made in the assay buffer (1:50000). Serial 2-fold dilutions of sera (previously heat inactivated 30′ 56°C) were prepared by adding 25 μl of pre-diluted sera (starting from 1/4 dilution) to 25 μl of assay buffer previously added to the wells, mixing, and then transferring 25 μl into the next well (the procedure was repeated from column 2 to 10) (Figure 4C).

The assay was performed in sterile 96-well plates in a final volume of 50 μl per well, as represented in Figure 4D. Bacteria working dilution was added to each well (12.5 μl corresponding to 200 cfu). Then 12.5 μl of active complement were added to the wells in Columns 1 to 11, while heat inactivated complement was added in column 12 (Figure 4D). Indeed, two internal controls were introduced in the assay: a Complement Dependent Control, to measure the killing induced by the complement *per se* in absence of antibodies (column 11, Figure 4D) and a Complement Independent Control, to measure the killing induced by serum alone in presence of heat inactivated complement (column 12, Figure 4D).

Bacteria at time zero (T_0_) were plated in duplicate. The plate was incubated for 1 h at 37°C with 5% (v/v) CO_2_ in a humidified atmosphere and on a soft orbital rotating shaker. Bacteria at T_60min_ were sampled, and spotted onto agar plates (all wells were plated in duplicate). Agar plates were incubated overnight at 37°C in a humidified atmosphere with 5% (v/v) CO_2_. On the following day, colony forming units (cfu) were counted for T_0_ and T_60min_ in order to evaluate the bactericidal titer of each serum. The number of colonies in column 11 is the number of viable cells. We assumed that a serum dilution has bactericidal activity when after 1 h of incubation at 37°C, more than 50% of the bacteria were killed with respect to T_0_. The bactericidal antibody titer was expressed as the reciprocal of the final serum dilution yielding ≥50% killing at T_60min_ as compared with the average for control wells (T_0_).

## Additional file

Additional file 1: Table S1. Oligonucleotides used in this study.

## Abbreviations

NTHi: Non-typeable *Haemophilus influenzae*
SBA: Serum bactericidal assay
OM: Otitis media
COPD: Chronic obstructive pulmonary disease
