# Supplementary material for: Development of a serological assay to predict antibody bactericidal activity against non-typeable Haemophilus influenzae
Source: BMC Microbiol. 2015 Apr 18;15:87. doi: 10.1186/s12866-015-0420-x (PMC4409741; doi:10.1186/s12866-015-0420-x)
Supplement: Additional file 1: Table S1. — Oligonucleotides used in this study. [file 12866_2015_420_MOESM1_ESM.pdf]

Table S1

Protein	Oligo	Oligo sequence
HtrA	HtrA-for	ACTTTACCAAGTTTTGTTTCG
	HtrA-rev	TTGCACTAATAAATAGAAATTACTGT
OMP26	OMP26-for	GAAGAAAAAATTGCTTTCATTAATG
	OMP26-rev	TTTTTTCTCTTGTGCTTTTTCA
P6	P6-for	AGTTCATCTAACAACGATGCT
	P6-rev	GTACGCTAACACTGCACG
Protein D	Protein D-for	AGCAGCCATTCATCAAATATG
	Protein D-rev	TTTTATTCCTTTTAAGAATTCCACG
Protein E	Protein E-for	TCTGCTCAAATCCAAAAGG
	Protein E-rev	TTTTTTATCAACTGAAAATGCTT
PilA	PilA-for	ACTAAAAAAGCAGCGGTATCTGA
	PilA-rev	TTGTGTGACACTTCCGCAA
